# In silico analysis of the Val66Met mutation in BDNF protein: implications for psychological stress

**DOI:** 10.1186/s13568-024-01664-w

**Published:** 2024-01-22

**Authors:** Muhammad Adnan Shan, Muhammad Umer Khan, Warda Ishtiaq, Raima Rehman, Samiullah Khan, Muhammad Arshad Javed, Qurban Ali

**Affiliations:** 1 Center for Applied Molecular Biology, University of the Punjab, Lahore, Pakistan; 2 Institute of Molecular Biology and Biotechnology, The University of Lahore, Lahore, Pakistan; 3 Department of Plant Breeding and Genetics, Faculty of Agricultural Sciences, University of the Punjab Lahore, Lahore, Pakistan

**Keywords:** *BNDF* V66M, Stress, Mutation, Proteins stability, Stress-related disorders

## Abstract

The brain-derived neurotrophic factor (BDNF) involves stress regulation and psychiatric disorders. The Val66Met polymorphism in the *BDNF* gene has been linked to altered protein function and susceptibility to stress-related conditions. This in silico analysis aimed to predict and analyze the consequences of the Val66Met mutation in the *BDNF* gene of stressed individuals. Computational techniques, including ab initio, comparative, and I-TASSER modeling, were used to evaluate the functional and stability effects of the Val66Met mutation in BDNF. The accuracy and reliability of the models were validated. Sequence alignment and secondary structure analysis compared amino acid residues and structural components. The phylogenetic analysis assessed the conservation of the mutation site. Functional and stability prediction analyses provided mixed results, suggesting potential effects on protein function and stability. Structural models revealed the importance of *BDNF* in key biological processes. Sequence alignment analysis showed the conservation of amino acid residues across species. Secondary structure analysis indicated minor differences between the wild-type and mutant forms. Phylogenetic analysis supported the evolutionary conservation of the mutation site. This computational study suggests that the Val66Met mutation in *BDNF* may have implications for protein stability, structural conformation, and function. Further experimental validation is needed to confirm these findings and elucidate the precise effects of this mutation on stress-related disorders.

## Introduction

Stress is a well-known risk factor for psychiatric illnesses, such as depression and anxiety disorders (McEwen and Gianaros 2011). Stress is a primary determinant impacting students' psychological and physical well-being globally (Haider et al. 2023). However, individuals display a range of stress reactions that are impacted by hereditary and environmental factors (Gillespie et al. 2009; Lupien et al. 2009). The processes underlying stress regulation and the therapy of various conditions can be clarified by understanding the relationship between susceptibility genes and stress. A growth factor known as the brain-derived neurotrophic factor (BDNF) is thought to be a key player in stress-related neural alterations in the hippocampus and prefrontal cortex, including atrophy, cell death, and suppression of neurogenesis (Magarinos et al. 2011). The hypothalamic–pituitary–adrenal (HPA) axis is essential for controlling stress responses, and both animal and human research have shown that dysregulated HPA axis activity is linked to several illness problems. Numerous studies have emphasized the significance of adaptive brain and body responses to acute and chronic stress for preserving the best possible physical and mental health (McEwen 2008).

The most prevalent neurotrophic in the brain, brain-derived neurotrophic factor (BDNF), is a 27-kDa polypeptide that is essential for the survival, differentiation, and growth of brain neurons. It is important for use-dependent plasticity mechanisms like long-term potentiation, learning, and memory (Schinder and Poo 2000). It produces the BDNF protein, which regulates neuron survival, development, mood modulation, synapse formation, neurotransmitter balance, and neuroprotection. (Sakhawat et al. 2023) The *BDNF* gene on chromosome 11p13 harbours a functional single nucleotide polymorphism (SNP) referred to as rs6265 or Val66Met. This genetic variation leads to substituting valine (Val) with methionine (Met) at codon 66 of the proBDNF protein. The Val66Met SNP has demonstrated effects on the intracellular transportation and activity-dependent release of BDNF demonstrated effects on the intracellular transportation and activity-dependent release of (Egan et al. 2003; Dempster et al. 2005; Szeszko et al. 2005; PERVAIZ et al. 2021; GOHAR et al. 2023). The Val66Met SNP has a significant influence on BDNF secretion and function. Met allele carriers exhibit reduced BDNF secretion and impaired intracellular trafficking, affecting synaptic plasticity and neuronal survival (Park et al. 2017; Shen et al. 2018). The link between the Val66Met SNP and psychiatric disorders has been established by research. It has been connected to a higher incidence of illnesses like schizophrenia, anxiety, and depression. Different bioinformatics tools were used for the analysis of mutation (Chu et al. 2022; Farcas et al. 2023; Hassan et al. 2023; Ullah et al. 2023; Zahid et al. 2023). Particularly, the Met allele is frequently linked to an increased vulnerability to certain illnesses (Shen et al. 2018). Cognitive function is also affected by the Val66Met SNP. Met allele carriers may have altered cognitive abilities, including memory and learning impairments. Some studies suggest a connection between the Val66Met SNP and neurodevelopmental disorders like autism spectrum disorder (ASD). The Met allele has been associated with a higher risk of ASD (BASHIR et al. 2023).

The sorting protein sortilin interacts with BDNF in the prodomain area, and the Met alteration may decrease sortilin's ability to bind to BDNF (Chen et al. 2005a, b). This SNP has been connected to several psychiatric disorder symptoms, including mood, anxiety, and cognition. Interestingly, the prevalence of the Val66Met SNP varies among ethnic groupings, with Asians showing a higher frequency than Caucasians (Shimizu et al. 2004). For BDNF to be processed and transported properly within neurons, sortilin and BDNF must interact (Chen et al. 2005a, b). This relationship can be impaired, which can have far-reaching effects on neurological processes and possibly even contribute to diseases like depression. (Colucci-D’Amato et al. 2020).

It has been discovered that under some circumstances, the interactions between Sortilin and BDNF can be compromised. Sortilin interacts with proBDNF and controls the activity-dependent release of BDNF in cortical neurons, processing and transporting BDNF into neurons (Ho et al. 2019). However, it has been discovered that sortilin truncation mutations disrupt the sorting of BDNF to particular vesicles, impairing its appropriate transport and release (Szarowicz et al. 2022). One of the several circumstances that can lead to the disruption of the sortilin-BDNF interaction is the presence of Met alteration in the prodomain area of BDNF, which has been shown to decrease sortilin's ability to bind to BDNF. (Covaceuszach et al. 2022) In a previous study, the V66M polymorphism was induced by extended molecular dynamics simulations on a 3D ab initio generation model and homology modelling, which influenced the proBDNF pro-fundamental domain's movements, hydrogen-bonding network, and local and non-local secondary structure conformation. These factors can lead to disruption in molecular binding of BDNF, to its potential targets such as sortilin. (De Oliveira et al. 2019). Another similar structural bioinformatics and molecular dynamics simulation study pointed out the V66M mutation to induce instability in hproBDNF-HAP1 and hproBDNF-Sortilin1 complexes. (Zamani et al. 2019). This mutation that affects the binding capacity or sorting function of sortilin can lead to the disruption of its interaction with BDNF.

BDNF is essential for promoting the growth and development of neurons (Bathina and Das 2015). Impaired interaction with Sortilin can lead to decreased BDNF trafficking to the appropriate neuronal sites, resulting in reduced neuronal growth and development. BDNF plays a critical role in regulating synaptic activity and plasticity. When BDNF is unable to properly interact with Sortilin, it can lead to alterations in synaptic transmission and plasticity, which can negatively impact neuronal communication and function (Ho et al. 2019). BDNF is also important for promoting neuronal survival and protecting neurons from damage and degeneration. Impaired interaction with Sortilin can lead to decreased BDNF signaling, which may compromise the survival of neurons and increase their vulnerability to various insults (Baydyuk and Xu 2014).s

Two theoretical frameworks, the diathesis-stress model (Caspi et al. 2003) and the differential-susceptibility hypothesis (Belsky et al. 2009), can be used to explain how the BDNF Val66Met polymorphism interacts with stressful environmental circumstances. According to the diathesis-stress paradigm, those with "vulnerability genes" are more likely to develop psychopathology when faced with environmental hardship. The differential-susceptibility hypothesis, on the other hand, contends that "plasticity genes" rather than vulnerability genes are what regulate the connection. This theory postulates that people with particular gene variations display differences in their developmental plasticity and vulnerability (Belsky and Pluess 2009). Environmental changes impact fewer "plastic" people, whereas more "plastic" people are more sensitive to both good and negative environmental effects.

The expression of BDNF in the prefrontal cortex (PFC) and hippocampus (HPC) has been ssshown to decrease in response to stress (Taliaz et al. 2011). On the other hand, it has been discovered that BDNF treatment, whether through peripheral or central routes, can lessen the behavioral effects of persistent, unexpected, mild stress (Schmidt and Duman 2010). These results imply that BDNF may be important in controlling stress reactions and stress-induced behaviors. Studies with BDNF heterozygous knock-out mice (BDNF ±) subjected to prolonged solitary stress have identified a significant depressive-like behavioral pattern(Duman et al. 2007). A synergistic connection between stress and BDNF deficiency was also highlighted in the forced swim test (FST), where male BDNF ± mice exposed to mild stress from handling and repeated saline injections displayed longer periods of immobility(Advani et al. 2009). The study examined how the V66M mutation affected BDNF. Functional and stability consequences were highlighted through computational simulations and predictions. The mutation was discovered to occur at a non-conserved location, validating a trustworthy theoretical model of BDNF. Molecular dynamics simulations revealed modifications to secondary structure, hydrogen bonds, and fundamental movements. These results imply that the V66M mutation may affect *BDNF* function, presumably related to psychiatric illnesses (De Oliveira et al. 2019).

Another research was to determine how the *BDNF* Val66Met polymorphism affected individuals' reactions to a social stress test. A substantial interaction between *BDNF* genotype, sex, and cortisol response was found in the results, demonstrating gender differences. While female val/val homozygotes had the lowest cortisol surge, male val/val homozygotes showed a larger rise. These results imply that the BDNF polymorphism affects stress reactivity in men and women differently, perhaps resulting in gender-specific reactions to social stress(Shalev et al. 2009). Long-term exposure to work-related stress can cause depression, and *BDNF* polymorphism may play a significant role in this process. The research was done to better understand the interaction between chronic stress and the *BDNF* Val66Met variation that results in depression in Chinese healthcare professionals. The findings highlight the complex relationship between inherited traits and occupational stress in the context of mental health in this particular population, and they show that this gene-stress interaction has a significant impact on depressive symptoms in the workforce (He et al. 2018). Researchers found that the *BDNF* Val66Met polymorphism significantly affected the association between stress and depression in a meta-analysis of 31 trials with 21,060 participants. The study focused on how early adversity and stressful life experiences interact with the Met allele concerning depression. These results emphasize the *BDNF* Val66Met polymorphism's moderating function in the stress-depression association (Zhao et al. 2018). In Pakistan, research dedicated to molecular approaches for stress-related disorders has been scarce. As a response, this in silico study is crafted to predict and analyze the significance of the Val66Met mutation in the *BDNF* gene.

## Materials and methods

### Database and gene sequence retrieval

The UniProt database (UniProt ID: P23560) was used to obtain the sequences and details for the BDNF protein and its variant V66M. The given FASTA sequence retrieved the *BDNF* gene sequence (accession number: CAA62632.1).

### Structural modeling

Three-dimensional structures of BDNF were produced using both ab initio and comparative modeling techniques. Ab initio modeling, which predicts the protein's functional characteristics using computational methods and physical principles, was carried out using the AlphaFold (Jumper et al. 2021) program. The Swiss Model (Waterhouse et al. 2018)software for comparative modeling was used to establish the structure of BDNF using known protein structures that are homologous to it. The model with the highest validation scores demonstrated a strong agreement with experimental data and was chosen for further examination. The I-TASSER (Zhang et al. 2018) tool was also used to conduct TASSER modeling, which produced various BDNF structural models. The top five models were picked based on cluster size, which represents the similarity of projected structures, and given C-scores, which show the model's confidence level.

### Templates used in comparative modeling:

#### BNDF

Q4L0Y3.1.A Brain-derived neurotrophic factor.

AlphaFold DB model of BDNF_SPECI (gene: BDNF, organism: Spermophilus citellus (European suslik) (Citellus citellus)).

#### BNDF V66M

Q7YRB4.1.A Brain-derived neurotrophic factor.

AlphaFold DB model of BDNF_CANLF (gene: BDNF, organism: Canis lupus familiaris (Dog) (Canis familiaris)).

### Structure validation

Several validation tools were applied to ensure the excellence and durability of the chosen BDNF structure. ERRAT(Caimano et al. 2014) scores were assigned to both the wild-type and mutant BDNF structures; higher scores signify higher quality. The stereochemical quality of the structures was evaluated using Procheck (Laskowski et al. 1996) analysis, which assesses several geometric parameters. ProSa (Wiederstein and Sippl 2007) and Verify-3D (Lüthy et al. 1992) scores were generated to evaluate the agreement between the 3D models and the relevant amino acid sequences.

### Secondary structure analysis

The GOR-IV(Vasfi Marandi et al. 2018) was used to examine the secondary structures of both wild-type and mutant BDNF structures. The objective was to compare the two forms' structural components (such as beta sheets and alpha helices).

### Post-mutation functional and stability prediction

Several techniques were used to forecast how the V66M mutation in the BDNF protein would affect its stability and functionality. First, the functional significance of the Val66Met mutation was evaluated using the PhD-SNP (Capriotti et al. 2006) tool for SNP prediction analysis. PolyPhen-2 (Adzhubei et al. 2013) was employed to forecast the functional effects of the mutation, giving information about potential effects on protein function. MUpro (Cheng et al. 2006) and MutPred2 (Pejaver et al. 2020) tools were both used to assess the effect on protein stability. These methods evaluate numerous protein characteristics and properties to ascertain the potential stability implications brought on by the mutation. Additionally, the SIFT (Vaser et al. 2016) method was used to foresee the precise impact of the Val66Met mutation on the BDNF protein. DDGun used evolutionary traits to predict modifications in protein stability brought on by mutations (Montanucci et al. 2019). DUET was used to predict the effects of mutation by calculating the change in folding eergy (G) post-mutation (Pires et al. 2014). mCSM (Pires et al. 2013) was used for predictions of protein stability, protein–protein interactions, and protein-DNA interactions. DynaMut was used to predict the effects of V66M mutation on protein dynamics and stability using normal mode analysis (Rodrigues et al. 2018). Further, MAESTROweb was used to detect protein stability changes upon mutation by measuring free energy shift (Laimer et al. 2016).

### Sequence alignment

The amino acid sequences of BDNF from various animals were compared using Clustal W (Larkin et al. 2007) and Clustal Omega (Sievers et al. 2011) for multiple sequence alignment. The objective was to determine whether the amino acid residues varied or were conserved between the species.

### Phylogenetic analysis

The evolutionary conservation level of each amino acid in BDNF was determined using the ConSurf (Ashkenazy et al. 2016) service. For the phylogenetic study, 150 reference sequences were chosen, the CSI-BLAST homologous search technique was used, Bayesian alignment was used, and MAFFT-L-INS-i calculations were performed. The investigation attempted to evaluate the amino acid's conservation at position 66 and identify whether it was buried or exposed within the protein structure.

This study contributes to a deeper understanding of the characteristics and possible effects of the mutation by using numerous computational techniques and algorithms.

## Results

To evaluate the effects of the Val66Met mutation in the BDNF protein, many functional and stability prediction analyses were carried out in the present study. The results of all the analyses are as follows:

### Structural modeling

This research used three different modeling techniques to create three-dimensional structures of the BDNF protein: ab initio modeling, comparison modeling, and the top five predictions from the I-TASSER model.

### Ab initio modeling

**Fig. 1:**
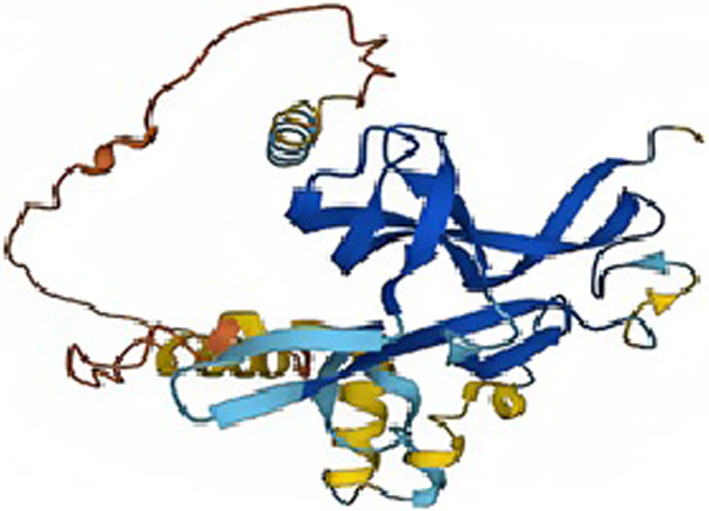
AlphaFold's structural model of the BDNF protein. AlphaFold's structural model of the BDNF protein is displayed in the form of a cartoon, as viewed in PyMOL. The 3D structure of BDNF was predicted using ab initio modeling utilizing the AlphaFold technique, which considered the amino acid sequence and physical laws

AlphaFold, a cutting-edge ab initio modeling technique, was utilized to predict the 3D structure of BDNF protein, as shown in Fig. 1. AlphaFold predicts the 3D coordinates of all heavy atoms in the protein's structure using the basic amino acid sequence as input. The resulting structure was subjected to various quality checks and validation, to determine the efficiency of predicted protein structure. The predicted structure can also be used to perform additional analysis regarding protein functionality.

**Fig. 2:**
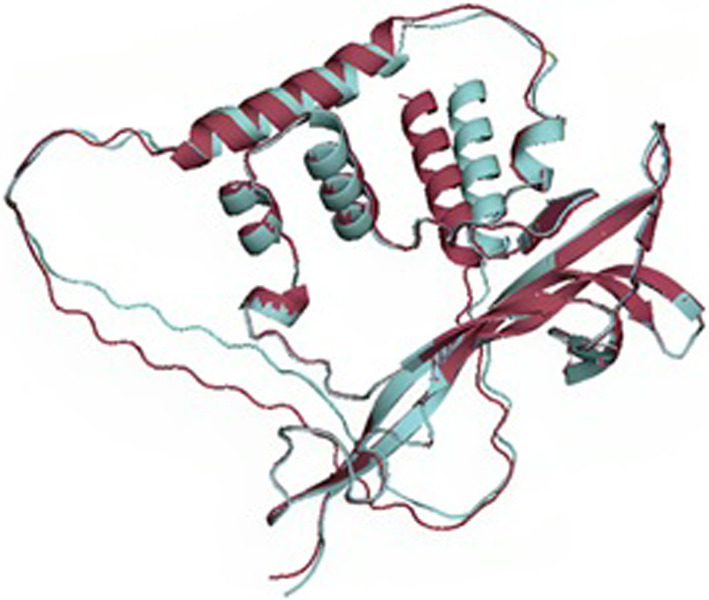
Superimposition of wild-type BDNF and mutated BDNF

The signaling molecule that initiates signal cascades below NTRK2 is a crucial component. Studies have shown in Fig. 2 that it activates the heterodimeric receptor of NGFR and SORCS2 to initiate signaling cascades (PubMed:24,908,487, PubMed:29,909,994). Long-term depression (LTD) and synaptic plasticity are regulated by the signaling pathway that NGFR and SORCS2 control. Interaction between SORCS2 and NGFR encourages neuronal death. In addition, it results in the collapse of neuronal growth cones, as observed in related biological processes.

According to research, this mutation can affect how BDNF works and how it interacts with NTRK2, also known as TrkB, a BDNF receptor. The V66M mutation has been linked to poorer fear memory in research on rodents, which is consistent with results from other BDNF mice models (Autry and Monteggia 2012). Additionally, this mutation has been linked to traits such as weight gain and neurobehavioral disorders as well as modifications in hippocampus structure and behavior (Sonoyama et al. 2020).

### Comparative modelling

The Swiss Model was used for the comparative model of both BNDF and BNDF V66M as shown in Fig. 2. Both structures were modeled by selecting the 3 best available templates that displayed maximum sequence identity. The 3 resultant 3D structures of BNDF and BNDF V66M, built comparatively to 3 selected templates for both the proteins, were analyzed based on GMQE score and sequence identity. For BNDF, the model with the highest GMQE (0.72) and a sequence similarity of 99.17% was selected for further analysis. Similarly, the BNDF V66M model with a sequence identity of 97.98% and GMQE score of 0.73 was selected.

### I-TASSER model of gene BDNF:

#### Prediction of top 5 I-TASSER model

**Fig. 3:**
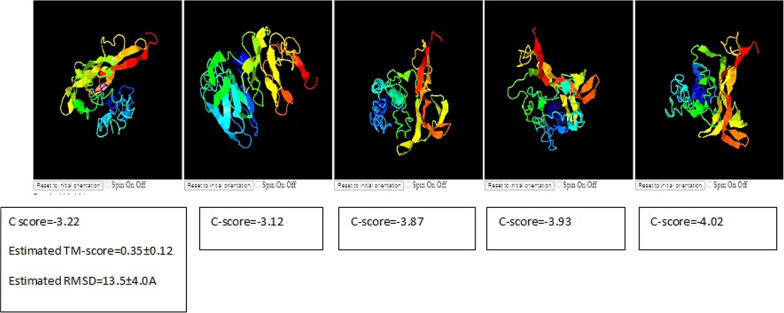
I-TASSER models of the BDNF gene. A number of the created structural conformations (decoys) were evaluated using the C-score for confidence. The top five models, which correspond to the biggest structural clusters, were chosen. To evaluate structural quality, the TM-score and RMSD values were computed. Due to simulation convergence, fewer clusters signify higher quality

The I-TASSER model, which created several structural conformations known as decoys, was used to analyze the BDNF gene. The SPICKER program clusters the decoys to choose the most trustworthy models. The top five models correlated to the biggest structure clusters and were identified and released. The C-score, which considers threading template alignments and the convergence parameters of the structure assembly simulations, was used to evaluate the confidence of each model. Higher values of the C-score denote greater confidence, with values ranging from − 5 to 2. The TM-score and RMSD values were calculated with the understanding that the C-score and protein length serve as indicators of structural quality. It is significant to notice that the original model frequently exhibits superior quality. However, infrequently, lower-ranked models have been shown to have higher C-scores. Benchmark testing has demonstrated that, in some cases, lower-ranked models may even exhibit superior quality than higher-ranked models. Less than five clusters could be formed if the I-TASSER simulations converge, which indicates high quality due to the simulations' convergence as shown in Fig. 3.

#### Structure validation

**Fig. 4:**
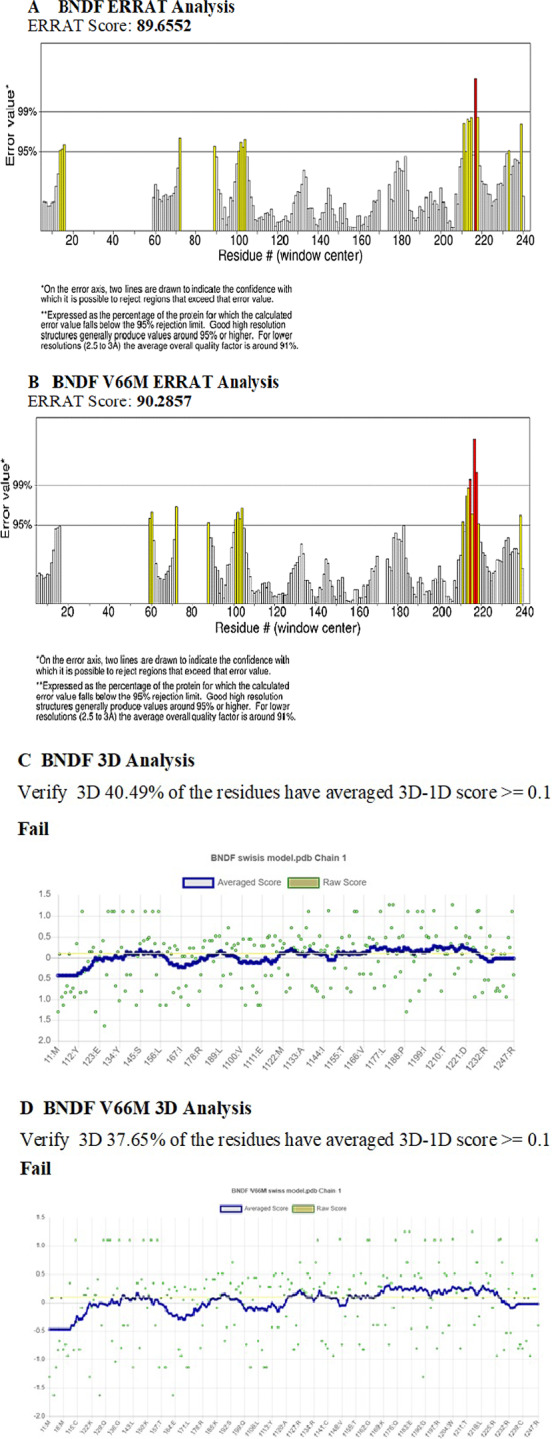

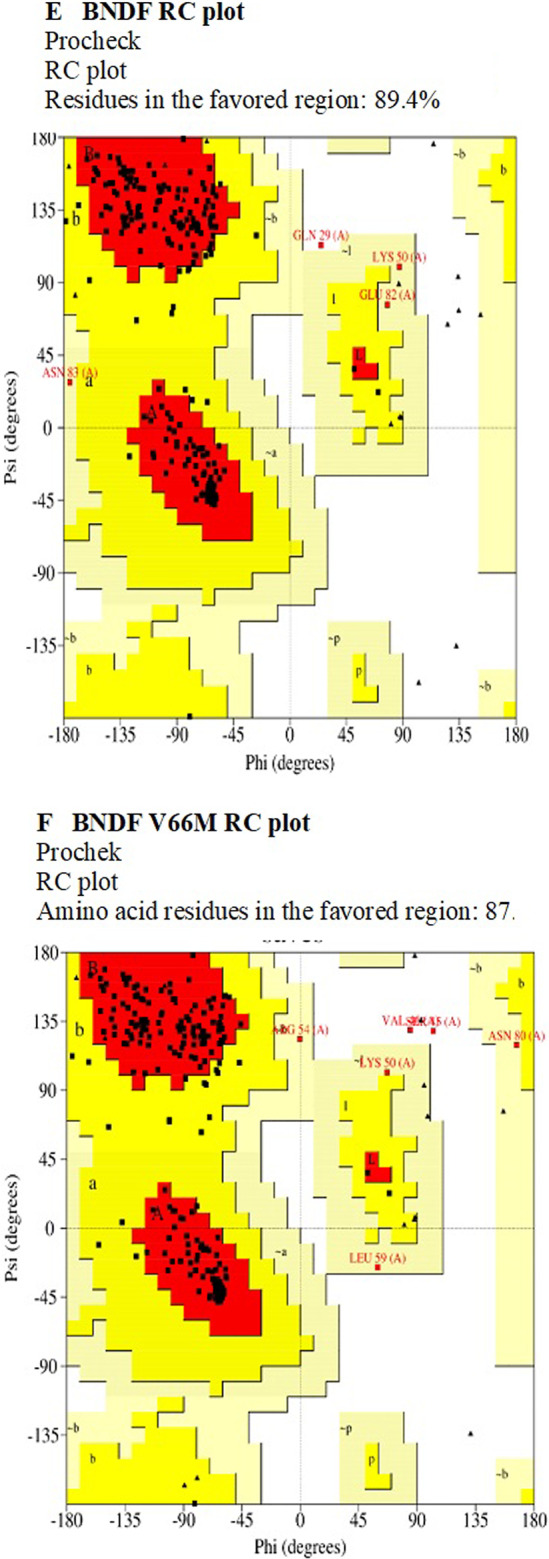
Validation results of BDNF protein models. **A** Wild-type BDNF structure: ERRAT score 89.6552. **B** Mutant BDNF (V66M) structure: ERRAT score 90.2857. **C** Wild-type BDNF: Verify3D 40.49% residues with 3D-1D score ≥ 0.1 (Fail). **D** Mutant BDNF (V66M): Verify3D 37.65% residues with 3D-1D score ≥ 0.1 (Fail). **E** Wild-type BDNF: 89.4% residues in the favored region by Procheck. **F** Mutant BDNF (V66M): 87.6% residues in the favored region by Procheck. These results validate the accuracy and stereochemical quality of the BDNF protein models

In the present investigation, the chosen structure of the BDNF protein, both in its wild-type and mutant form, was submitted to extensive structure validation utilizing various methods to evaluate the accuracy and dependability of the derived models. ERRAT scores were obtained for both constructions, with higher scores indicating higher quality as shown in Fig. 4.The ERRAT score for the wild-type BDNF structure was 89.6552, whereas the ERRAT score for the mutant BDNF (V66M) structure was just a hair higher at 90.2857. These results indicate that both structures are generally of good quality, with the altered structure showing a little higher score. These ratings imply that the produced structures accurately depict the BDNF protein.

**Fig. 5:**
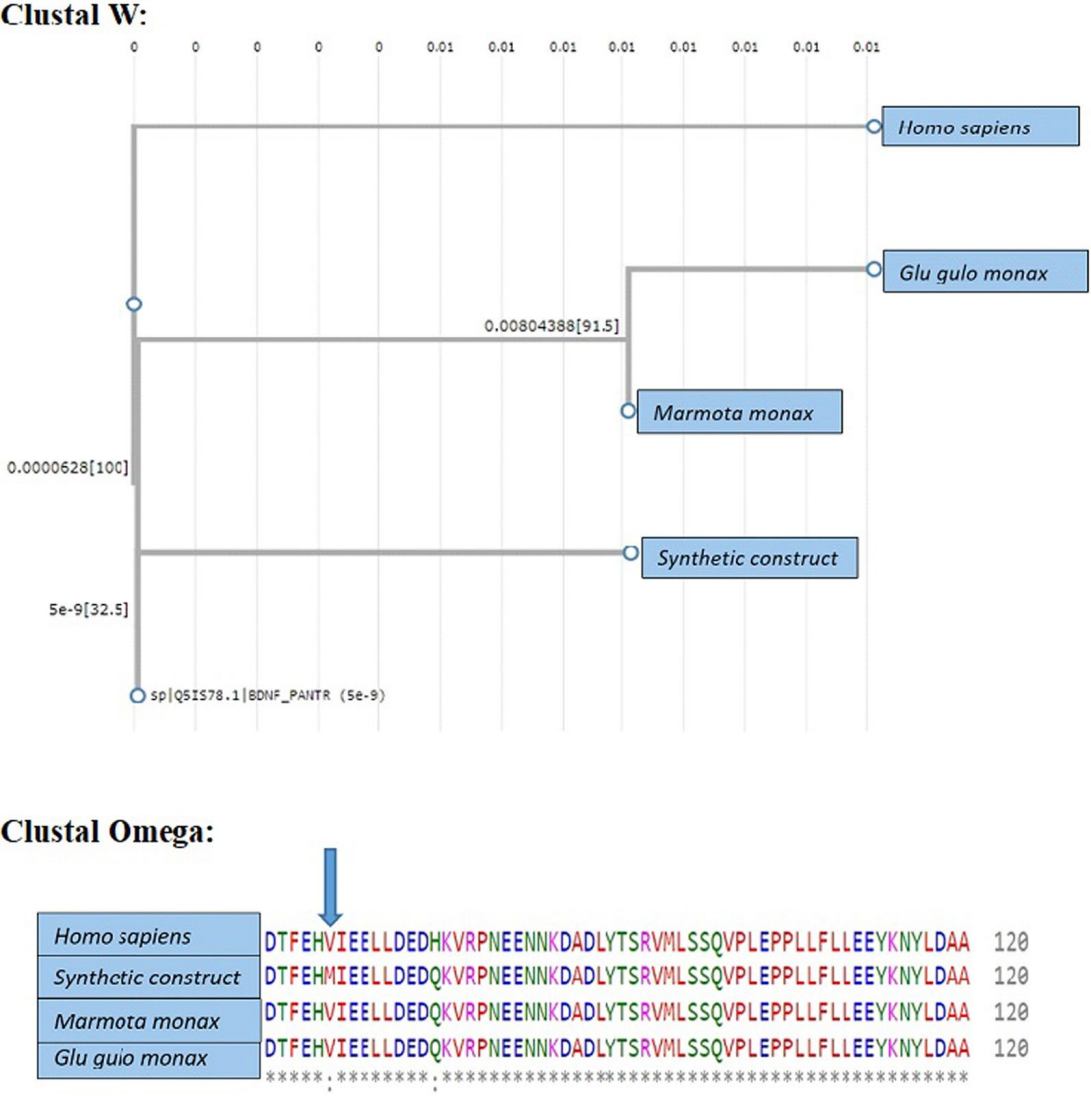
Sequence alignment analysis of the BDNF protein. There is significant conservation of amino acid sequence across species, as shown by multiple sequence alignment using Clustal W and Clustal Omega. The fact that there have been no changes to the amino acid composition shows how important the BDNF protein is to evolution and function

Procheck analysis was used to evaluate the stereochemical quality of the structures. For the wild-type BDNF structure, 89.4% of the residues were found in the Ramachandran plot's preferred area, indicating good stereochemical quality. For the altered BDNF structure, 87.6% of the amino acid residues were found in the preferred area; these results further attest to the high quality of the structures as a whole, even though the mutant structure showed a slightly lower proportion as mentioned in Fig. 5. To evaluate the degree of correspondence between the respective amino acid sequences and the 3D models, Verify3D scores were created (Fig. 4). It is asserted that the cutoff was missed, resulting in a "Fail" result, because the percentage of residues having an averaged 3D-1D score greater than or equal to 0.1 for both structures fell below it. This suggests that the predictions produced by the models and the properties of a well-folded protein may differ or be in conflict.

The ERRAT values indicate that both structures have strong overall qualities, with a marginally better score for the altered structure. The Verify3D scores, on the other hand, show variations from predicted protein folding properties, maybe pointing out places where the models need to be improved. Although the modified structure occupies the favored region of the Ramachandran plot with a somewhat lower proportion than the native structure, the favorable occupancy of residues there shows that both structures have good stereochemical quality. The interpretation and analysis of the BDNF protein's structural models within the study's context are greatly aided by these validation results.

#### Sequence alignment analysis

Clustal W and Clustal Omega were used for multiple sequence alignment to compare the amino acid sequences of the BDNF protein from different species. The main goal was to find any differences or resemblances in the amino acid residues between these species. According to the sequence alignment results using Clustal W and Clustal Omega, there have been no changes in the amino acid composition of BDNF in any of the three species examined as shown in Fig. 5. According to this discovery, several animals share a highly conserved amino acid sequence for BDNF. The amino acid sequence's conservation suggests that the BDNF protein has retained its evolutionary significance and functional importance.

#### Secondary structures

**Fig. 6:**
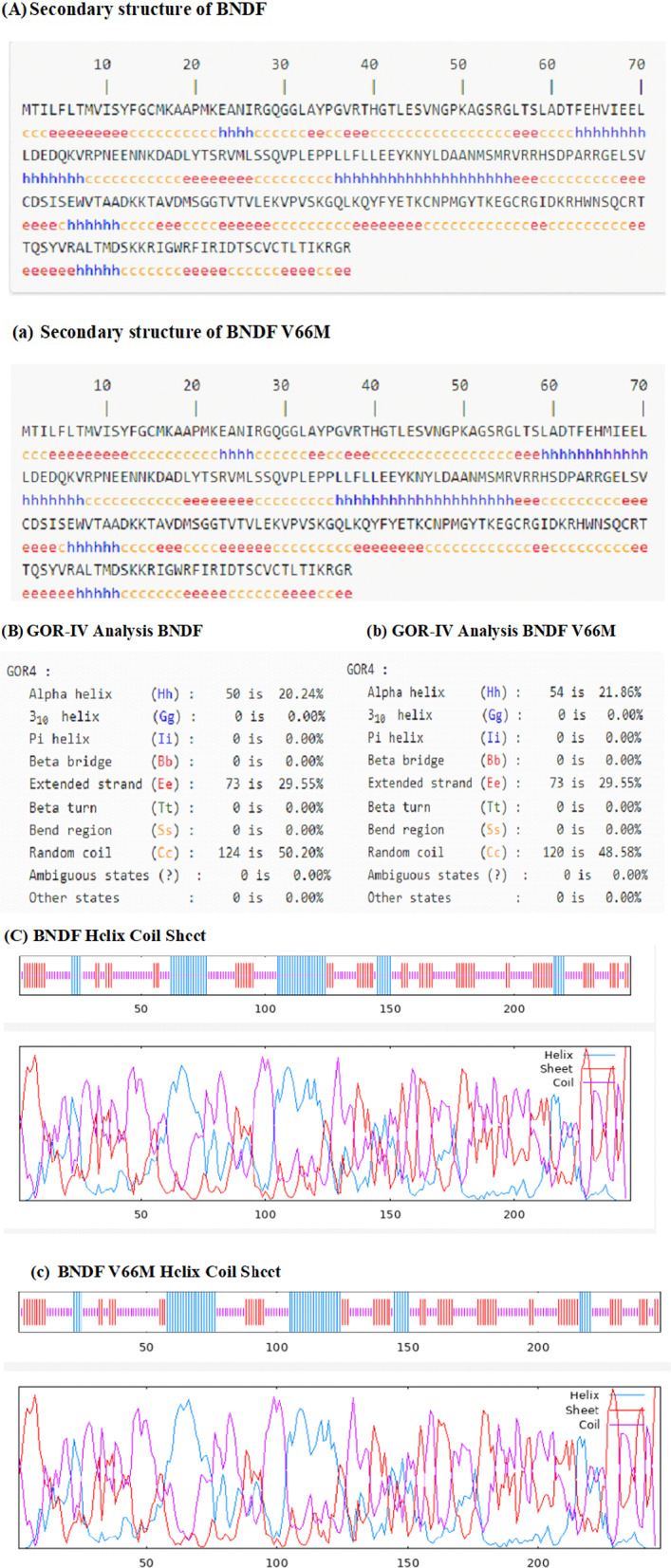
Comparative analysis of the secondary structure of BDNF and BDNF V66M using the GOR IV method. **A** Wild-type BDNF's secondary structure composition, displays alpha helices, extended strands, and random coil regions. **a** Secondary structure composition of BDNF V66M, showing largely identical structural elements. **B** Wild-type BDNF analysis, emphasizing the distribution of helices (20.24%), coils (50.20%), and sheets (29.55%). **b** The BDNF V66M shows a similar distribution of secondary structure components like the wild type with 21.86% helices, 29.55% sheets, and 48.58% coils. **C** Graphical representation of Wild-type BDNF's helix, coil, and sheets. **c** Graphical representation of the helix, coil, and sheet in BDNF V66M

The GOR-IV technique was used to analyse the secondary structure of both the wild-type and mutant BDNF structures (Fig. 6). The objective was to examine the structural components between the two forms, including beta sheets, alpha helices, and other secondary structure motifs.

The research findings showed that sections of random coils, extended strands, and alpha helices were present in the wild-type BDNF. The protein's structure is significantly influenced by alpha helices, which were expected to make up about 20.24% of the residues. An estimated 29.55% of the sequence comprises extended strands, which connect to neighbouring strands through intermolecular hydrogen bonds. Wild-type BDNF (50.20%) was mostly found in random coil regions, flexible segments devoid of recognizable secondary structure components. In the wild-type BDNF structure, no beta bridges, beta twists, or bend regions were observed. The secondary structure investigation of the mutant BDNF (V66M) revealed similarities to the wild-type structure, albeit with minor changes in the distribution of alpha helices and random coil regions. The mutant BDNF sequence was anticipated to contain 21.86% alpha helices and roughly 29.55% extended strands. A large portion (48.58%) of the mutant BDNF sequence was attributed to areas with random coils. Similar to the wild-type, the mutant BDNF did not generate 310 helices, pi helices, beta bridges, beta twists, or bend areas.

#### Functional and stability prediction analysis

To evaluate the effects of the Val66Met mutation in the BDNF protein, many functional and stability prediction analyses were carried out in the present study.

#### PhD-SNP prediction analysis

According to the PhD-SNP algorithm's predictions, the V66M mutation was tolerated with a score of 0.18. This shows that the mutation is unlikely to have a significant functional impact on the BDNF protein. The analysis also demonstrated a moderate level of sequence conservation, with a median conservation score of 3.09. This shows that the region of the mutation is reasonably conserved, with 43 sequences included in the sequences under analysis. In addition, the forecast highlighted the significance of Val66 since amino acids with a probability of 0.05 or less were considered potentially hazardous. These findings highlight the significance of Val66 in maintaining the protein's structure and functions.

#### PolyPhen-2 prediction

PolyPhen-2 was used to assess the effects of the Val66Met mutation on functioning. PolyPhen-2 scored the mutation with a value of 0.822, indicating it would likely be detrimental. As a result, there is a higher chance that the mutation will affect how the protein functions. The prediction's 0.93 specificity and 0.84 sensitivity values are further evidence that PolyPhen-2 is highly accurate in detecting potentially dangerous alterations.

#### MutPred2 prediction

The MutPred2 score was generated to assess the impact of the Val66Met mutation on protein stability. The mutation's predicted MutPred2 score was 0.322. When the MutPred2 score is higher, destabilization is more likely. This moderate score shows that the BDNF protein may become unstable due to the Val66Met mutation. This shows that the mutation may impact how stable the protein structure is.

#### SIFT analysis

The impact of the Val66Met mutation on the BDNF protein was assessed by the SIFT (Sorting Intolerant from Tolerant) approach. According to SIFT's study, the mutation is tolerated, as indicated by a value of 1. This finding strengthens the argument that the Val66Met mutation is unlikely to impact how proteins function significantly. The tolerance prediction suggests that substituting methionine for valine at position 66 may not negatively impact the BDNF protein's capacity to function correctly.

#### MUpro analysis

MUpro is frequently used to analyse how protein changes affect their stability. The Val66Met mutation may decrease the stability of the BDNF protein, according to the MUpro study. This implies that changing methionine to valine at position 66 might impact the BDNF protein's overall stability. Such a destabilizing action can change the structural makeup of the protein, which will then impact its functional characteristics. The MUpro study concludes that the BDNF protein's stability will probably decrease due to the Val66Met mutation. These combined functional and stability prediction analyses offer insightful information on the potential consequences of the Val66Met mutation in the BDNF protein. The findings show that the mutation may have various effects on protein function and stabilizing emphasizing the need for more experimental studies to confirm these hypotheses and clarify the specific functional ramifications of the mutation.

MUpro Analysis predicted both value and sign of energy change using a Support Vector Machine (SVM) and sequence information only. The predicted Delta delta G value was − 0.53414246 which indicates a decrease in the stability of the protein structure. A negative delta delta G suggests that the mutation results in decreased stability. The SVM method predicts an increase in the stability of the protein structure with a confidence score of 0.46814845. The neural network method also predicts an increase in the stability of the protein structure with a confidence Score of 0.6733155127013581. The V66M mutation is predicted to decrease protein structure stability, according to the recommended SVM analysis of delta G. While SVM and neural network methods suggest increased stability, the recommended method indicates a decrease.

#### DDGun analysis

Utilizing evolutionary characteristics, DDGun forecasted changes in protein stability resulting from mutations. Positive G values represent possible benefits, whilst negative G values indicate possible harm. The BNDF V66M G's value of − 0.3 indicates that the mutation may result in decreased stability.

#### DUET analysis

DUET calculates protein stability impact using folding-free energy change, potentially revealing functional alterations in proteins for illnesses or protein engineering. According to the DUET estimated stability change (G), the mutation in BNDF is expected to have destabilizing effects at a rate of − 0.18 kcal/mol.

#### mCSM analysis

Protein stability, interactions between proteins, and interactions between proteins and DNA can all be predicted with the help of mCSM. The mCSM Predicted Stability Change (G): − 0.3 kcal/mol predicts the destabilizing effects of the BNDF V66M mutation.

#### DynaMut analysis

The projected Gibbs free energy change following a mutation, as reported by DynaMut, was − 0.071 kcal/mol (Stabilizing). Normal mode analysis-based predictions gave the G ENCoM (Elastic Network Contact Model) value of − 0.004 kcal.mol-1.K-1, indicating a decrease in the flexibility of NBDF protein upon mutation.

**Fig. 7:**
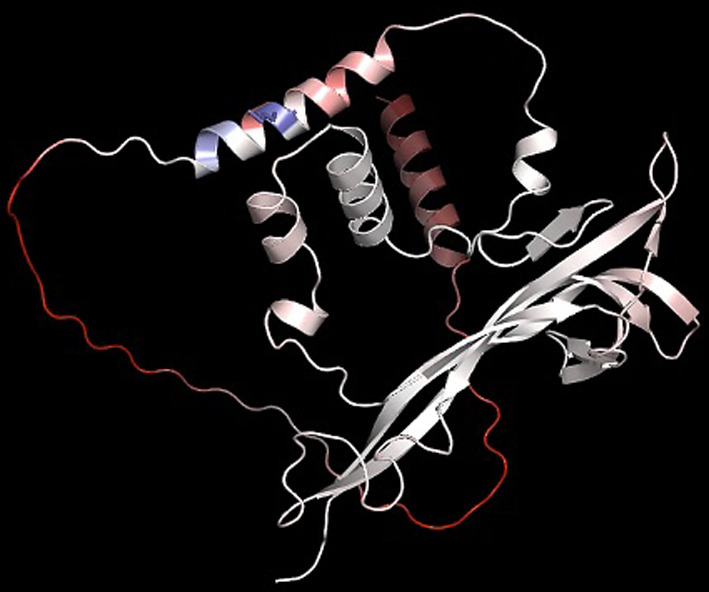
The vibratory entropy of the altered BNDF amino acids changed hue as a result of the mutation. A stiffer construction is represented by RED and one that is more flexible by BLUE

The difference in vibrational entropy energy between the wild-type and mutant forms of the protein, computed as SVib ENCoM, was found to be 0.184 kcal.mol-1.K-1, which suggests that the BNDF became more flexible after mutation, and might possess higher entropy as shown in Fig. 7.

**Fig. 8:**
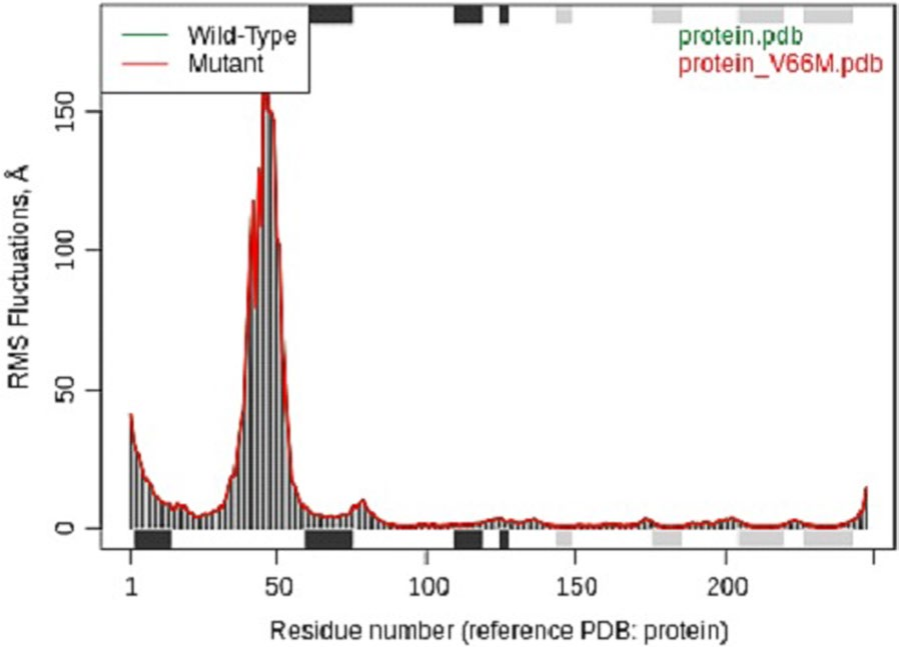
The sort of secondary structure (strands of black and grey helices) present in each sequence section accentuates the figure's top and bottom boundaries

Following their extraction from the corresponding 3D structures, the wild-type and mutant sequences were aligned. Below are the normal mode data results for each of the sequences (Fig. 8).

#### MAESTROweb

By measuring free energy shift and contributing to the detection of destabilizing mutations, it provides significant insights for protein engineering and study. A stabilizing mutation is indicated by the overall anticipated change in stability or G value, predicted by MAESTROweb for the substitution of G in place of D is − 0.617 kcal/mol. Additionally, it was discovered that the confidence estimation, or cpred was 0.869 which is extremely reliable.

#### Phylogenetic analysis

**Fig. 9:**
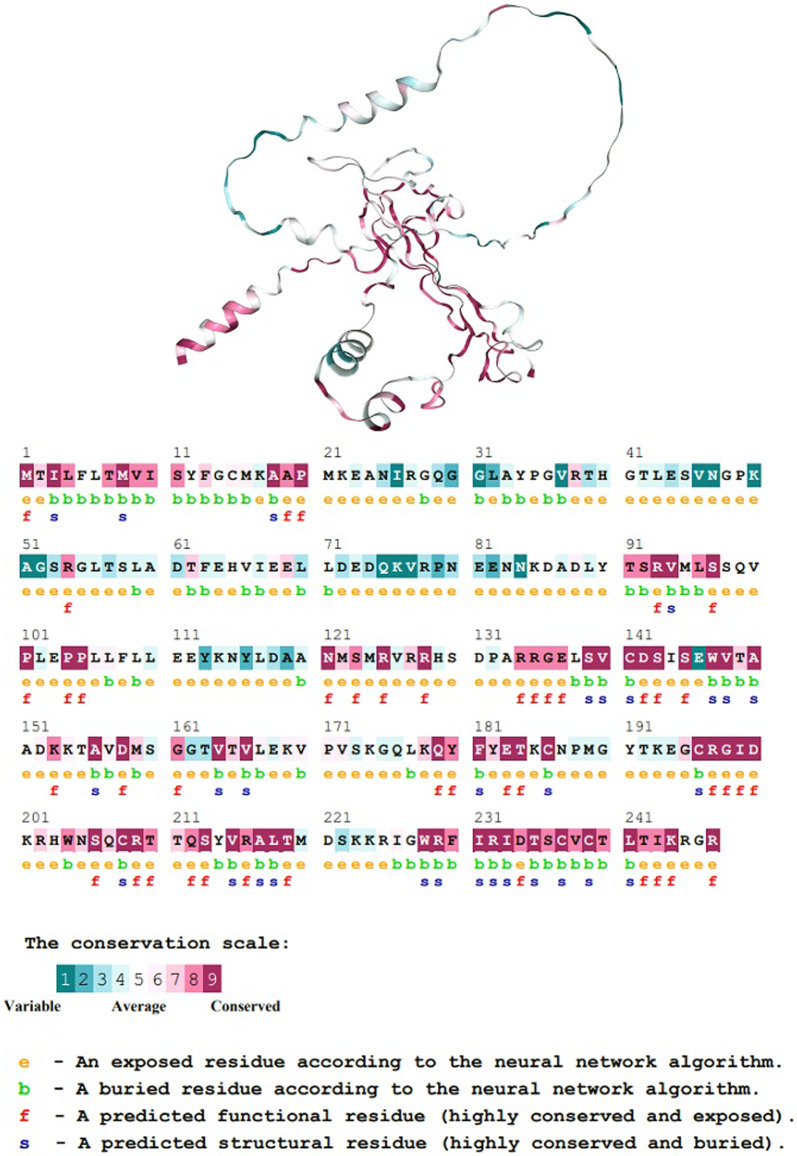
Phylogenetic analysis of BDNF. Phylogenetic analysis of BDNF amino acid conservation shows moderate conservation of the V66M mutation at position 6, indicating its functional or structural relevance. The residue's function in preserving protein stability and intermolecular interactions is supported by the prediction that it will be buried inside the protein structure

A phylogenetic analysis using the ConSurf software was used to evaluate the evolutionary conservation of each amino acid in BDNF as shown in Fig. 9. Numerous parameters and techniques, including the homologous search algorithm (CSI-BLAST), multiple iterations, the E-value cut-off, the protein database (UNIREF-90), the choice of reference sequences, sequence identity thresholds, the Bayesian alignment approach, and the evolutionary substitution model, were employed in the investigation.

The V66M mutation at position 6 of BDNF is an averagely conserved amino acid, according to the findings of the phylogenetic analysis. This implies that a particular residue has been preserved during evolution, indicating functional or structural relevance. A further prediction made by the neural network algorithm was that the residue at position 6 would be buried within the protein structure. This suggests it is inside the protein and less likely to be exposed to the solvent environment. The amino acid at position 6 has been conserved, suggesting that it is important to preserve the BDNF protein's overall structure or functionality. Its putative function in protein stability or intermolecular interactions is further supported by the fact that this residue was buried.

## Discussion

Understanding the impacts of specific protein mutations on physiological functioning is a critical area of genetic research. Brain-derived neurotrophic factor (BDNF) is a neurotrophic factor highly expressed in the brain and is involved in maintaining, sustaining, and promoting neurogenesis in particular brain regions. It is linked to the consequences of stress on the brain and plays a significant role in mental health disorders, such as depression and anxiety. Previous research has proven that stress and altered BDNF expression in significant brain areas are related (Miao et al. 2020). This computational investigation examined how the BDNF protein's Val66Met variation altered its structural and functional properties, as well as its possible implications in stressed people. We predicted and analyzed the implications of this mutation using various computational techniques, including functional and stability prediction analyses, structural modelling, structure validation, sequence alignment, secondary structure analysis, and phylogenetic analysis, offering insightful information about its potential effects. The combination of various computation tools helped to improve the automated prediction accuracy and significantly outperform just relying on the results from a single tool.

The structure of proteins is crucial for understanding their functions and disease-related alterations (Dorn et al. 2014) Theoretical modelling offers an affordable and efficient way of predicting protein structures in addition to sequencing techniques (Krebs and De Mesquita 2016). Our structural modelling methods such as ab initio modelling, comparison modelling, and I-TASSER models, helped elucidate the three-dimensional structures of both wild-type and mutant BDNF. The selection of the best obtained 3D model of wild and mutated BDNF was based on the structural validation results using established methods like ERRAT, Procheck, and Verify3D. Later, these methods were also employed for the comparative validation of the selected models of BDNF and BDNF V66M, to evaluate the plausible structural changes that might take place in case of this mutation. This structural variation is also backed up by various studies such as a distinct experiment employing molecular dynamics simulations and homology modelling revealed that the V66M polymorphism affects both secondary structure conformation and significant motions (De Oliveira et al. 2019). The 3D models of BDNF and BDNF V66M overall displayed good quality. The comparison of wild and mutated structures only revealed slight differences in the V66M mutation. Similarly, the secondary structure analysis also suggested that V66M mutation does not significantly alter BDNF’s secondary structural elements. The alpha helices, extended strands, and random coil areas were all present in the wild-type BDNF, although they were distributed slightly differently in the mutant BDNF than in the wild-type. In a study by Steif and colleagues (Sneha et al. 2017), the impact of the Val66Met mutation on the stability of the protein's alpha helix was highlighted. This study investigated the impact of the Val66Met mutation on BDNF, crucial in mood disorders' pathophysiology. Using computational tools, it revealed structural alterations: a decline in alpha-helix content and increased random coils, potentially leading to protein deformation. These findings shed light on the mutation's potential role in affecting BDNF function, offering insights into mood disorder susceptibility linked to this genetic variant.

The predicted nonsignificant structural alterations post-mutation suggest that the mutation might have occurred in a way that preserves the overall three-dimensional conformation of the protein. The extent to which a protein's structure is affected by a mutation depends on the specific mutation and its location within the protein. Contrarily, another detailed structural investigation also showed that the V66M mutation makes protein complexes unstable (Zamani et al. 2019). Sequence alignment analysis using Clustal W and Clustal Omega revealed that the amino acid composition of BDNF is conserved across different mammals which in turn suggests that this specific sequence is necessary for its functional role.

The post-mutation functional and structural predictions presented contrasting results. The results obtained through PhD-SNP, SIFT, and MAESTROweb classified this mutation to be stabilizing and tolerated. While all other analyses predicted the V66M replacement as destabilizing. This vast contrast overall suggests the potentially deteriorating impact of this mutation, which is also evident from prior studies.

The discrepancy between the SIFT and MUpro analyses regarding the impact of the Val66Met mutation on the BDNF protein can be attributed to the different aspects of protein function that each algorithm focuses on. Using sequence homology, the bioinformatics tool SIFT (Sorting Intolerant from Tolerant) determines if an amino acid alteration at a specific location in a protein would impact its function. SIFT indicated that the Val66Met mutation is tolerable in this instance, indicating that it is unlikely to have a substantial effect on the BDNF proteins functionality. However, MUpro is a tool for examining how modifications to proteins impact their stability. It predicts how a mutation will alter a protein & its stability using sequence data and a Support Vector Machine (SVM). In this case, the MUpro analysis predicted that the Val66Met mutation may decrease the stability of the BDNF protein, which could impact its functional characteristics.

Its important to note that both functional and stability predictions are valuable in understanding the potential consequences of a mutation. While SIFT suggests that the mutation may not significantly impact the function of the BDNF protein, MUpro suggests that it may decrease the stability of the protein, which could in turn impact its functional characteristics. Furthermore, the discrepancy between the SIFT and MUpro analyses highlights the complexity of understanding the effects of mutations on protein function and stability. Both types of predictions offer valuable insights, and further experimental studies are needed to confirm and clarify the specific functional ramifications of the Val66Met mutation in the BDNF protein.

The phylogenetic analysis performed by ConSurf software provided information on the evolutionary conservation of each amino acid in BDNF. The moderate conservation of the Val66 residue suggests that it might be significant structurally or functionally. The prediction that this residue is buried inside the protein structure provides additional support for its likely involvement in protein stability or intermolecular interactions.

In a similar context, a different study using in silico prediction methods showed that proBDNF and its V66M version are expected to contain naturally disordered areas (Kailainathan et al. 2016; Sneha et al. 2017). These results add to our understanding of the structural effects of the BDNF Val66Met mutation and raise the possibility that disordered areas may be involved in the functional effects.

The computational research suggests that the Val66Met mutation in the BDNF protein may impact the protein's stability, structural conformation, and overall function. These results require further investigation and experimental validation to understand the precise consequences of this mutation on stress-related diseases.

Reduced BDNF function is linked to the V66M mutation. Neural plasticity and mood control both benefit from BDNF. According to studies, those who carry the V66M mutation may be more susceptible to the negative consequences of stress. The brain may find it more difficult to adjust to stimuli if BDNF function is reduced. There is evidence connecting this mutation to a higher incidence of mood disorders such as anxiety and depression. Stress can make these symptoms worse for people who have the V66M mutation.

The extensive exploration of the Val66Met mutation within the BDNF protein unveiled a complex landscape of its potential impacts. Employing diverse analyses from structural modelling to functional predictions, this study revealed a nuanced understanding. While the mutation seems generally well-tolerated concerning the protein's overall structure and function, the prediction analyses presented a mixed perspective, indicating both potential destabilization and tolerance. Notably, the conservation of the mutated amino acid position and similarities in secondary structures underscored its moderate impact. However, the study highlights the necessity for further experimental research to precisely elucidate the mutation's functional consequences. Despite these complexities, this investigation lays a robust foundation, offering invaluable insights into the mutation's potential effects on BDNF, fostering pathways for future inquiries into its significance in human health and disease.

## Data Availability

The produced, collected, or generated during the study has been given in the manuscript file.

## Abbreviations

BDNF: The brain-derived neurotrophic factor
FST: Forced swim test
HPA: Hypothalamic–pituitary–adrenal
HPC: Hippocampus
LTD: Long-term depression
NGFR: Nerve Growth Factor Receptor
NTRK2: Neurotrophic Tyrosine Kinase Receptor 2
PFC: Prefrontal cortex
RC: Ramachandran plot's
RMSD: Root Mean Square Deviation
SNP: Single nucleotide polymorphism
SORCS2: Sortilin-Related VPS10 Domain Containing Receptor 2
SIFT: Sorting Intolerant from Tolerant
TASSER: Threading/ASSEmbly/Refinement
TM-score: Template Modeling score
V66M: Val66Met
